# S05-1 Online tool to stimulate physical activity for older people with dementia - development and dissemination

**DOI:** 10.1093/eurpub/ckac093.023

**Published:** 2022-08-29

**Authors:** Liesbeth Preller

**Affiliations:** Knowledge Centre for Sport & Physical Activity Netherlands, Utrecht, The Netherlands

**Keywords:** physical activity, older persons, implementation, volunteers, participation

## Abstract

Physical activity (PA) stimulates physical health, quality of life, and potentially cognition in people with dementia. However, home dwelling older people with dementia are in general less active than peers. For those attending daycare, the amount of physical activity is highly dependent on the centre's policy. For those without daycare, municipalities are responsible for support but generally there is no attention to PA for this target group. Professionals and volunteers in day care centers, and informal caregivers lack awareness and knowledge to support the people with dementia. Figures on users of other online information on PA show that video's and practical information are highly rated.

As part of a research consortium with 4 scientific partners, we were funded for dissemination of research outcomes to practice and policy. We therefore decided to build an online tool with easy to understand instruction movies and background information on theme's like strong legs and physical activity friendly layout of spaces. Human movement scientists provided the technical exercise information. We tested the information on user-friendliness among professionals. The instrument was launched in the week of World Alzheimer's day 2019. The tool has been viewed often since then. We will present development and dissemination of the tool and will discuss ongoing optimization.

